# Analysis of mental health outcomes in major versus minor upper extremity amputations: a retrospective national database study

**DOI:** 10.1007/s00402-026-06319-y

**Published:** 2026-04-23

**Authors:** Victoria Nedder, Joyce Wang, Kacy Peek

**Affiliations:** Department of Orthopaedic Surgery, University of Virginia Health System, Charlottesville, United States

**Keywords:** Upper extremity amputation, Mental health, Antidepressants, Psychotherapy

## Abstract

**Background:**

Upper extremity amputations are associated with developing psychiatric conditions. The aim of this study is to identify differences in mental health outcomes for patients undergoing major versus minor upper extremity amputations.

**Methods:**

Data were obtained from the PearlDiver database between 2010 and 2022 using Current Procedural Terminology and International Classification of Diseases codes. Patients aged 10 and above without prior mental health diagnoses or antidepressant prescription records who underwent upper extremity amputations were stratified by major (shoulder disarticulation, arm, forearm, wrist, transmetacarpal) versus minor (single metacarpal, digit, phalanx) amputations. Demographic characteristics and rates of mental health diagnoses, antidepressant prescriptions, and psychotherapy care were assessed for 90 days and one year postoperatively, using Welch’s T-tests and Pearson’s chi-squared tests.

**Results:**

Patients with major amputations had increased risk of mental health diagnoses at 90 days (OR: 3.29, *p* < 0.001) and at one year (OR: 2.01, *p* < 0.001). Both groups had higher rates of mental health diagnoses than the general population. Patients undergoing major amputations had higher odds of starting antidepressants at 90 days (OR 3.82, *p* < 0.001) and at one year (OR 2.38, *p* < 0.001). Psychotherapy care was significantly increased after major amputations at 90 days (OR 5.47, *p* < 0.001) and at one year (OR 4.18, *p* < 0.001).

**Conclusions:**

Mental health disorders, antidepressant use, and psychotherapy care are significantly higher for major upper extremity amputations compared to minor amputations. Surgical teams should provide mental health resources to mitigate negative effects from mental health needs after upper extremity amputation.

**Level of evidence:**

III.

**Supplementary Information:**

The online version contains supplementary material available at 10.1007/s00402-026-06319-y.

## Introduction

Upper extremity amputations are significant, life-altering procedures. Upper extremity injuries are known to be associated with various effects on mental health [1, 2]. Amputations are associated with a higher risk of developing psychiatric conditions when compared to the general population [3–9] and compared to non-amputation upper extremity injuries [3]. Rates of psychiatric outcomes after upper extremity amputations vary significantly between studies, ranging between 16.4% and 67% [3, 4]. Furthermore, psychological factors are shown to affect functional outcomes in upper extremity injuries, highlighting the importance of identifying and addressing psychological distress in the recovery process [2, 5, 10, 11]. Psychological interventions, such as cognitive-behavioral therapy, may help individuals with psychological distress after upper extremity amputations improve their functional recovery and return to work [9, 12].

While prior studies have identified the association between upper extremity injuries and psychological distress, few studies have compared amputation types and their varying effects on mental health outcomes. Some studies have indicated that more proximal amputations have a higher risk of mental health disorders [3, 5], while others did not see a difference between amputation level [4]. Furthermore, most of the literature focuses on positive screening for mental health diagnoses, rather than the rates of patients actually pursuing psychological treatment. The aim of this study is to identify the difference in mental health outcomes and needs for patients undergoing minor versus major upper extremity amputations, including new mental health disorder diagnoses, use of psychotherapy care, and use of antidepressant medications.

## Materials and methods

This study was deemed exempt from Institutional Review Board (IRB) approval given that it was a secondary analysis of a Health Insurance Portability and Accountability Act (HIPAA)-compliant database containing deidentified patient records.

**Fig. 1:**
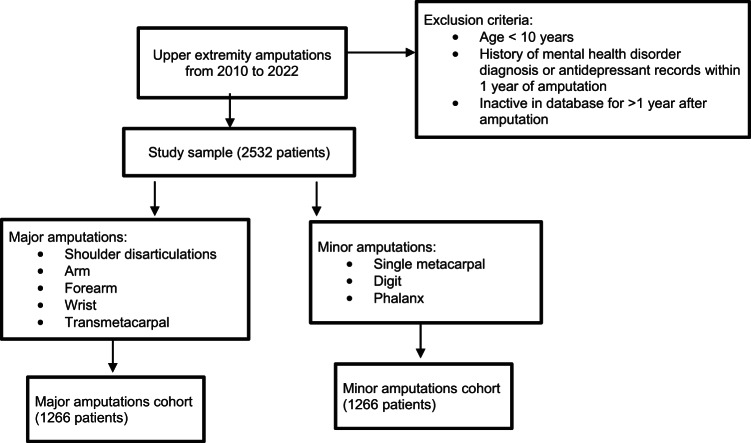
Patient selection criteria. Flowchart illustrating how patients were selected and sorted into major versus minor amputation groups

The M170 dataset in the PearlDiver (PearlDiver Inc., Colorado Springs, Colorado, USA) database was queried for first instances of upper extremity amputations from 2010 to 2022 using relevant Current Procedural Terminology (CPT) codes. Exclusion criteria included age younger than 10 years old, patients who had a history of mental health disorder diagnosis or antidepressant records within one year of amputation, and those who were inactive in the database for over one year after amputation. Major amputations included shoulder disarticulation, arm, forearm, wrist, and transmetacarpal amputations. Minor amputations included single metacarpal, digit, or phalanx amputations, as these amputations generally result in less cosmetic and functional hand differences [13, 14]. Figure 1 summarizes the patient selection process. The CPT code descriptions for the study cohorts are summarized in Supplemental Table S1. Eligible patients were matched one-to-one based on age range, gender, the Elixhauser Comorbidity Index (ECI), and smoking history.

The PearlDiver database was queried for ICD diagnosis codes of 10 mental health disorders in the major and minor amputation groups. Mental health disorders included depression, anxiety, sleep disorder, bipolar disorder, panic disorder, post-traumatic stress disorder (PTSD), suicidal ideation, suicide attempt, alcohol-related disorder, and drug-related disorder, excluding alcohol. Primary outcomes were new diagnoses of mental health disorders, new claims of psychotherapy care, and antidepressant use within 90 days and one year post-amputation. The list of International Classification of Diseases (ICD)−9, ICD-10 codes, and medication names used to define the study population and outcome variables is detailed in Supplemental Table S2. Pearson χ2 and Welch’s t-test were performed to compare between-group differences. Adjusted odds ratios (ORs) and 95% confidence intervals (CIs) were obtained from multivariate logistic regression models controlling for age range, gender, ECI, and smoking history. All statistical analyses were performed using the R statistical software package in PearlDiver, with significance defined a priori as *p* < 0.05.

## Results

After matching, we analyzed data from 2532 patients who underwent upper extremity amputations. Of those, 1266 patients underwent major amputations, and 1266 had minor amputations. Table 1 demonstrates relevant demographic data for both groups.

**Table 1:** Demographic information of matched patients who underwent major versus minor upper extremity amputations

Characteristics	Major Amputations	Minor Amputations	*P*-value
	*N* = 1226 (50%)	*N* = 1226 (50%)	
Age (mean ± SD)	51.6 ± 18.1	51.7 ± 18.2	0.981
Sex (female)	779 (63.5%)	779 (63.5%)	1
Tobacco Use	141 (11.5%)	141 (11.5%)	1
ECI (mean ± SD)	5.0 ± 4.3	5.0 ± 4.3	1

The average age of patients undergoing major amputations was 51.6 versus 51.7 for minor amputations. Both major and minor amputation groups had a slightly higher percentage of women (63.5%) compared to men. Both groups had similar prevalence of tobacco use and equivalent ECIs. There were no significant differences between groups based on these demographic variables (*p* > 0.05 for all).

Tables 2 and 3 summarize the differences between the major amputation and minor amputation cohorts for rates of mental health disorders, antidepressant usage, and psychotherapy use at 90 days and one year postoperatively.

**Table 2:** Comparisons of new diagnoses of mental health disorders, antidepressants, and psychotherapy use within 90 days post-amputation

90-Day Outcomes	Major Amputations		Minor Amputations		Univariate *p*-value	Multivariate Adjusted OR (95% CI)	Multivariate *p*-value
	*N* = 1226		*N* = 1226				
**Mental Health Disorders**	227	18.5%	85	6.9%	< 0.001	3.29 (2.52–4.34)	< 0.001
**Mood Disorders** Depressive Disorder	86	7.0%	25	2.0%	< 0.001	4.10 (2.59–6.77)	< 0.001
Bipolar Disorder Suicidal Ideation Suicide Attempt **Anxiety Disorders** Anxiety Disorder Panic Disorder **Other Disorders**	5 7 3 64 1	0.4% 0.6% 0.2% 5.2% 0.1%	3 2 0 25 2	0.2% 0.2% 0.0% 2.0% 0.2%	0.723 0.182 0.248 < 0.001 1.000	3.00 (1.86–5.02)	< 0.001
Sleep Disorder	38	3.1%	9	0.7%	< 0.001	4.88 (2.38–11.32)	< 0.001
Post-Traumatic Stress Disorder **Substance-Related Disorders**	40	3.3%	3	0.2%	< 0.001	13.79 (4.98–57.20)	< 0.001
Alcohol-Related Disorder	24	2.0%	11	0.9%	0.041	2.18 (1.09–4.65)	0.034
Drug-Related Disorder, Excluding Alcohol	52	4.2%	31	2.5%	0.026	1.70 (1.08–2.71)	0.024
**Antidepressant Use**	144	11.7%	45	3.7%	< 0.001	3.82 (2.70–5.53)	< 0.001
SSRI	61	5.0%	27	2.2%	< 0.001	2.51 (1.58–4.11)	< 0.001
SNRI	51	4.2%	9	0.7%	< 0.001	5.90 (3.03–12.91)	< 0.001
TCA	30	2.4%	7	0.6%	< 0.001	5.08 (2.25–13.60)	< 0.001
Atypical Antidepressant	9	0.7%	3	0.2%	0.148		
Serotonin Modulators	37	3.0%	15	1.2%	0.003	2.68 (1.47–5.16)	0.002
MAOi	0	0.0%	0	0.0%	N/A		
**Psychotherapy**	37	3.0%	8	0.7%	< 0.001	5.47 (2.57–13.50)	< 0.001

SSRI: Selective Serotonin Reuptake Inhibitors; SNRI: Serotonin/Norepinephrine Reuptake inhibitors; TCA: Tricyclic Antidepressants; MAOi: Monoamine Oxidase Inhibitors

**Table 3:** Comparisons of new diagnoses of mental health disorders, antidepressants, and psychotherapy use within one year post-amputation

One Year Outcomes	Major Amputations		Minor Amputations		Univariate *p*-value	Multivariate Adjusted OR (95% CI)	Multivariate *p*-value
	*N* = 1226		*N* = 1226				
**Mental Health Disorders**	358	29.2%	217	17.7%	< 0.001	2.01 (1.65–2.45)	< 0.001
**Mood Disorders** Depressive Disorder Bipolar Disorder Suicidal Ideation Suicide Attempt **Anxiety Disorders**	161 15 15 3	13.1% 1.2% 1.2% 0.2%	77 7 8 0	6.3% 0.6% 0.7% 0.0%	< 0.001 0.134 0.209 0.248	2.45 (1.83–3.31)	< 0.001
Anxiety Disorder Panic Disorder **Other Disorders**	110 2	9.0% 0.2%	66 6	5.4% 0.5%	< 0.001 0.288	1.80 (1.31–2.50)	< 0.001
Sleep Disorder	71	5.8%	29	2.4%	< 0.001	2.71 (1.74–4.33)	< 0.001
Post-Traumatic Stress Disorder **Substance-Related Disorders**	67	5.5%	11	0.9%	< 0.001	6.47 (3.53–13.03)	< 0.001
Alcohol-Related Disorder	44	3.6%	29	2.4%	0.096		
Drug-Related Disorder, Excluding Alcohol	101	8.2%	84	6.9%	0.221		
**Antidepressant Use**	261	21.3%	131	10.7%	< 0.001	2.38 (1.89–3.02)	< 0.001
SSRI	117	9.5%	73	6.0%	0.001	1.71 (1.26–2.34)	< 0.001
SNRI	93	7.6%	35	2.9%	< 0.001	2.89 (1.95–4.39)	< 0.001
TCA	64	5.2%	26	2.1%	< 0.001	2.65 (1.67–4.32)	< 0.001
Atypical Antidepressant	33	2.7%	22	1.8%	0.173		
Serotonin Modulators	72	5.9%	35	2.9%	< 0.001	2.16 (1.44–3.32)	< 0.001
MAOi	0	0.0%	0	0.0%	N/A		
**Psychotherapy**	63	5.1%	18	1.5%	< 0.001	4.18 (2.44–7.56)	< 0.001

SSRI: Selective Serotonin Reuptake Inhibitors; SNRI: Serotonin/Norepinephrine Reuptake inhibitors; TCA: Tricyclic Antidepressants; MAOi: Monoamine Oxidase Inhibitors

The most diagnosed disorder in the major amputation group at 90 days and at one year was depression, followed by anxiety. For the minor amputation group, the most common at 90 days was drug-related disorder, followed by depression and anxiety, and at one year was drug-related disorder, followed by depression. At 90 days, patients with major amputations had significantly increased odds of mental health diagnoses compared to minor amputation patients (OR: 3.29; 95% CI: 2.52–4.34; *p* < 0.001), with 18.5% of the major amputation cohort having a new diagnosis compared to 6.9% in the minor amputation cohort. These disparities persisted at one year, with rates of 29.2% in the major amputation cohort and 17.7% in the minor amputation cohort (OR: 2.01; 95% CI: 1.65–2.45; *p* < 0.001). Of note, increased odds of developing depressive disorder, anxiety disorder, sleep disorder, and PTSD observed in major amputation patients at 90 days persisted at one year post-amputation. However, the differences in developing alcohol-related disorder (OR: 2.18; 95% CI: 1.09–4.65; *p* = 0.034) and drug-related disorders (OR: 1.70; 95% CI: 1.08–2.71; *p* = 0.024) at 90 days did not persist at one year post-amputation in the major amputation group.

Antidepressant usage also varied among major versus minor amputation groups. Antidepressants included SSRIs, SNRIs, TCAs, atypical antidepressants, and serotonin modulators. Patients undergoing major amputations had higher odds of starting an antidepressant by 90 days at a rate of 11.7% compared with 3.7% of the minor amputations group (OR 3.82; 95% CI 2.70–5.35; *p* < 0.001). This significant difference persisted at one year, with a rate of 21.3% in the major amputation group versus 10.7% in those with minor amputations (OR 2.38; 95% CI 1.89–3.02; *p* < 0.001). The most used antidepressant in both groups at 90 days and at one year was SSRIs.

Psychotherapy use was also significantly increased after major amputations (3.0%) compared to minor amputations (0.7%) at 90 days (OR 5.47; 95% CI 2.57–13.5; *p* < 0.001). This disparity again persisted at one year, with prevalence of psychotherapy after major amputations at 5.1% compared with 1.5% in minor amputation patients (OR 4.18; 95% CI 2.44–7.56; *p* < 0.001).

## Discussion and conclusions

In this study, we described the differences in mental health diagnoses, antidepressant use, and psychotherapy use in patients undergoing major versus minor upper extremity amputations. Previous studies on the mental health outcomes of patients with upper extremity amputations have not separated major versus minor amputations and directly compared outcomes between these groups. Past studies also have not specifically looked at patients pursuing antidepressant or psychotherapy use after amputation, and rather only identified the prevalence of positive diagnoses.

Amputations and injuries to the hand and upper extremity have a significant effect on patients’ livelihoods and self-perception. Individuals use and rely on their hands for their activities of daily living, their vocations, and their identity; loss of part or all of the upper extremity can have deleterious effects on emotional well-being and sense of self [2]. Therefore, understanding and characterizing mental health outcomes in upper extremity amputation patients is vital.

We identified that the rates of mental health disorder diagnoses are higher in those with major compared to minor amputations at both 90 days and at one year. The rate of mental health diagnosis in major amputees was 18.5% at 90 days and 29.2% at one year, while that in minor amputees was 6.9% at 90 days and 17.7% at one year. The overall rate of mental health disorders in the general population was found to be 9.7% within the same time period this data was queried from.^3^ Both major and minor upper extremity amputees in our cohort therefore had higher rates than the general population during this time period. Dussik et al. identified that the incidence of multiple mental health disorders, including depression, psychosis, mood disorders, alcohol or opioid abuse, PTSD, and suicidality was higher for patients undergoing upper extremity amputation compared to the general population. They found a rate of 16.4% for mental health diagnoses, compared to 9.7% in the general population [3]. Shue et al. found similarly high rates of mental health disorders in upper extremity amputees, most commonly depression, with a rate of mental health diagnoses in their cohort at 67% [4]. Cohen-Tanugi et al. found 51% of upper extremity amputees screened positive for depression and 69% for PTSD [5]. Armstrong et al. found rates of 55.4% for depression and 23.4% for PTSD [6]. Our study aligns with this prior data, indicating high rates of depression after upper extremity amputations. Depression was the most commonly diagnosed condition in major amputees; however, minor amputations had drug-related disorder diagnoses as the most common. Our measured rates of mental health diagnoses were on the low side compared to prior studies, but still within the large range previously identified in the literature. This finding is likely due to our exclusion of prior mental health diagnoses, which is a risk factor for limb amputation, in order to identify new diagnoses of mental health disorders after upper extremity amputation.

Dussik et al. also found that patients with amputations proximal to the digits exhibited increased rates of psychiatric conditions compared to those limited to the digits or metacarpals [3]. Cohen-Tanugi et al. found similar trends toward higher rates of mental health diagnoses for more proximal amputations [5], while Shue et al. saw no significant differences based on level of amputation [4]. Our study adds to these discrepancies by finding that major amputations have significantly higher rates of mental health conditions compared to minor amputations. In contrast to prior studies that grouped amputations based on location, we further characterized amputations as major or minor based on the extent of the amputation, for example, with transmetacarpal amputations being major and single metacarpal, digit, or phalanx amputations being minor. This adds to the literature by providing a clearer distinction between types of amputations that may be more or less likely to contribute to mental health diagnoses.

This study also identifies the use of antidepressants and psychotherapy in patients after upper extremity amputation, which prior studies have not assessed. We found that rates of antidepressant use and psychotherapy are increased in patients with major amputations compared to minor amputations, which persisted at both 90 days and at one year. This indicates that not only are these patients screening positive for mental health conditions, but that they also are requiring and pursuing psychological intervention for their symptoms. It is possible that those with minor amputations are receiving fewer referrals for treatment of psychological symptoms, which could contribute to lower antidepressant and psychotherapy usage, but the fact that rates of mental health diagnoses are significantly different between the two groups likely indicates that the need for antidepressants and psychotherapy differs between the groups as well. Rates of antidepressant use in the general population have been cited to be roughly 11% [15], while psychotherapy use in the general population has ranged from 6.2 to 10.3%, increasing in the past 20 years [16]. In this study, major upper extremity amputees had a rate of antidepressant use of 21.3%, greater than the general population, while minor amputees had a rate of 10.7%, similar to the general population. Psychotherapy use was lower in both major and minor amputation groups (5.1% and 1.5%, respectively), compared with the general population rates of 6.2 to 10.3%. These findings could again be confounded by the availability of these resources to patients during their recovery, as it is interesting that upper extremity amputation patients in this study had significantly higher rates of mental health diagnoses compared to the general population but seemed to be using psychotherapy less than the general population. This highlights the need for increased referrals and resources made available to patients after amputations.

These findings are paramount because several prior studies have identified that mental health disorders are consistently associated with functional impairment after upper extremity injury [2, 5, 10, 11]. By identifying patients at risk for or developing mental health conditions, earlier interventions could help mitigate negative outcomes. Studies have shown that psychological interventions such as cognitive behavioral therapy can help improve patient function after upper extremity injury, including return to work [12]. Coping strategies were also shown to be associated with psychological distress after upper extremity amputation, highlighting the role that psychological intervention could play on improving psychological distress [9]. However, there are several barriers to incorporating mental health care into orthopedic practice. One study found that while 98% of orthopedic surgeons believed mental health resources were important for patients, 71% never or rarely take mental health history at intake appointments [17]. Potential barriers to addressing mental health concerns included time, as well as dedicated clinic personnel to screen for these disorders [18]. Other barriers include the fact that patients themselves need to follow-up and pursue the psychiatric referral, which may not always occur [18]. Incorporating education on the importance of mental health, both for providers and patients, could greatly improve both the frequency of screening and the follow-through of patients engaging with these referrals [17, 18]. Including mental health surveys as part of intake documentation and having social work personnel available in clinic could help incorporate consideration of mental health into clinic flow more seamlessly [18]. Kulkarni et al. describe a clinical workflow and outline that would be ideal for management of patients after upper extremity amputations, including screening for mental health diagnoses, on-site psychologists to meet with patients with concerning screenings, group therapy sessions, and peer mentorship to aid in recovery [19]. However, access to mental health resources for patients is significantly limited by insurance status [17, 18, 20, 21]. This remains a large barrier to care, and future work needs to be dedicated to increasing accessibility of mental health care for all patients to truly have an impact on outcomes.

Timing of diagnosis of mental health conditions is important for upper extremity amputation patients. Cohen-Tanugi et al. found that the average first date of positive screening after upper extremity amputation was 6 months for depression and 10 months for PTSD, but was highly variable between patients [5]. Our study found that rates of mental health diagnoses varied between the 90 day and one year mark, with higher rates at one year compared to 90 days for both major and minor amputation groups. This highlights the need for ongoing monitoring of patients post-amputation to ensure that they are being screened for mental health conditions and have access to resources, especially if symptoms may not be apparent for several months or more after injury or surgery. This indicates the importance of identifying patients at higher risk for mental health diagnosis. Patients may be lost to follow-up as they get further out from surgery, which can lead to missing these diagnoses if patients are no longer attending follow-up appointments. Major amputations are at higher risk for development of mental health diagnoses, although minor upper extremity amputations still are associated with higher rates of mental health diagnoses compared to the general population as well. Given this information, there should be closer monitoring of both major and minor upper extremity amputation patients in longer-term follow-up to allow for screening for mental health conditions. Resources in psychiatry and psychology are limited, and knowing which patients are at higher risk, specifically major upper extremity amputees and to a lesser degree minor upper extremity amputees, allows physicians to target those resources prophylactically toward these individuals. It is important to note that although connection to psychiatry or psychology resources cannot prevent mental health conditions, it can help ensure that these issues are appropriately addressed to improve patient outcomes, maintain quality of life, and improve recovery.

This study has several limitations. First, underlying indication for amputation was not considered. It is possible that different pathologies leading to amputation (for example traumatic amputation versus amputation for malignancy, infection, vascular disease, or other) may lead to different mental health outcomes. We also did not assess whether socioeconomic status and access to care had any impact on these numbers. It is possible that the lower rates of mental health diagnoses and usage of psychotherapy and antidepressants seen in the minor amputation group could be confounded by a lack of referral for or ability to seek out resources that would allow for such diagnoses or therapies. Also, as this was a retrospective study relying on database information, inaccuracies in reporting or coding for mental health diagnoses and therapy usage can skew the results. Furthermore, we used general epidemiological studies to identify rates of mental health diagnoses, antidepressant use, and psychotherapy use in the general population to compare to our data. It is possible these numbers do not reflect a sample exactly like ours. For example, the population used for this study is an insured population. Research has shown that insurance status impacts usage of mental health resources, indicating that our data may be biased by using an entirely insured population [17, 18, 20, 21].

In conclusion, upper extremity amputations can have a significant effect on patients’ mental health and well-being. We identified that the risk of mental health diagnosis after upper extremity amputation is higher than the general population, and higher for those with major compared to minor amputations, with the effect persisting at both 90 days and one year. Antidepressant use and psychotherapy use were significantly higher at 90 days and one year after major amputations compared to minor amputations, although antidepressant usage in patients with minor amputations and psychotherapy usage in both major and minor amputation groups were lower than rates for the general population. Understanding that patients with upper extremity amputations, and specifically major amputations, are at higher risk of needing treatment for psychological distress, and that psychological distress can negatively impact function and recovery, we can better target psychiatric resources towards this population to mitigate negative effects that may come from mental health conditions after upper extremity amputation.

## Supplementary Information

Below is the link to the electronic supplementary material.

Supplementary Material 1

## Data Availability

Our data was internally saved to a secure desktop but can be available for review upon request.
