# Supplementary material for: Analysis of mental health outcomes in major versus minor upper extremity amputations: a retrospective national database study
Source: Arch Orthop Trauma Surg. 2026 Apr 23;146(1):158. doi: 10.1007/s00402-026-06319-y (PMC13106270; doi:10.1007/s00402-026-06319-y)
Supplement: Supplementary file 1 — Supplementary Material 1 [file 402_2026_6319_MOESM1_ESM.docx]

Supplemental Table S1.

| **Minor Amputations** | **Code Descriptions** |
| --- | --- |
| CPT-26235 | Partial excision (craterization, saucerization, or diaphysectomy) bone (eg, osteomyelitis); proximal or middle phalanx of finger |
| CPT-26236 | Partial excision (craterization, saucerization, or diaphysectomy) bone (eg, osteomyelitis); distal phalanx of finger |
| CPT-26910 | Amputation, metacarpal, with finger or thumb (ray amputation), single, with or without interosseous transfer |
| CPT-26951 | Amputation, finger or thumb, primary or secondary, any joint or phalanx, single, including neurectomies; with direct closure |
| CPT-26952 | Amputation, finger or thumb, primary or secondary, any joint or phalanx, single, including neurectomies; with local advancement flaps (V-Y, hood) |
| **Major Amputations** | **Code Descriptions** |
| CPT-24900 | Amputation, arm through humerus; with primary closure |
| CPT-24920 | Amputation, arm through humerus; open, circular (guillotine) |
| CPT-24930 | Amputation, arm through humerus; re-amputation |
| CPT-24931 | Amputation, arm through humerus; with implant |
| CPT-25900 | Amputation, forearm, through radius and ulna |
| CPT-25905 | Amputation, forearm, through radius and ulna; open, circular (guillotine) |
| CPT-25909 | Amputation, forearm, through radius and ulna; re-amputation |
| CPT-25920 | Disarticulation through wrist |
| CPT-25922 | Disarticulation through wrist; secondary closure or scar revision |
| CPT-25924 | Disarticulation through wrist; re-amputation |
| CPT-25927 | Transmetacarpal amputation; |
| CPT-25929 | Transmetacarpal amputation; secondary closure or scar revision |
| CPT-25931 | Transmetacarpal amputation; re-amputation |
| CPT-23900 | Interthoracoscapular amputation (forequarter) |
| CPT-23920 | Disarticulation of shoulder |

Supplemental Table S2.

| **Outcomes** | **Codes** |
| --- | --- |
| Depressive Disorder | ICD-9-D-311, ICD-10-D-F329, ICD-9-D-3004, ICD-10-D-F341, ICD-9-D-29632, ICD-10-D-F331, ICD-9-D-29620, ICD-10-D-F329, ICD-9-D-29630, ICD-10-D-F339, ICD-9-D-29633, ICD-10-D-F332 |
| Anxiety Disorder | ICD-9-D-30000, ICD-10-D-F419, ICD-9-D-30002, ICD-10-D-F411 |
| Sleep Disorder | ICD-9-D-78052, ICD-10-D-G4700, ICD-9-D-30742, ICD-10-D-F5101 |
| Bipolar Disorder | ICD-9-D-29680, ICD-10-D-F319 |
| Post-Traumatic Stress Disorder (PTSD) | ICD-9-D-30981, ICD-10-D-F4310, ICD-9-D-30928, ICD-10-D-F4323 |
| Panic Disorder | ICD-9-D-30001, ICD-10-D-F410 |
| Suicide Attempt | ICD-9-D-E9500:ICD-9-D-E9589, ICD-10-D-X71:ICD-10-D-X83, ICD-10-D-T1491 |
| Suicidal Ideation  Alcohol-Related Disorder  Drug-Related Disorder, Excluding Alcohol | ICD-9-D-V6284, ICD-10-D-R45851  ICD-9-D-291, ICD-9-D-2910, ICD-9-D-2911, ICD-9-D-2912, ICD-9-D-2913, ICD-9-D-2914, ICD-9-D-2915, ICD-9-D-2918, ICD-9-D-29181, ICD-9-D-29182, ICD-9-D-29189, ICD-9-D-2919, ICD-9-D-3030, ICD-9-D-30300, ICD-9-D-30301, ICD-9-D-30302, ICD-9-D-30303, ICD-9-D-3039, ICD-9-D-30390, ICD-9-D-30391, ICD-9-D-30392, ICD-9-D-30393, ICD-9-D-3050, ICD-9-D-30500, ICD-9-D-30501, ICD-9-D-30502, ICD-9-D-30503, ICD-9-D-5710, ICD-9-D-5711, ICD-9-D-5712, ICD-9-D-5713, ICD-9-D-7903, ICD-9-D-V113, ICD-9-D-V791, ICD-10-D-F1010, ICD-10-D-F10159, ICD-10-D-F10180, ICD-10-D-F10181, ICD-10-D-F10182, ICD-10-D-F10188, ICD-10-D-F1020, ICD-10-D-F1021, ICD-10-D-F10229, ICD-10-D-F10231, ICD-10-D-F10239, ICD-10-D-F10259, ICD-10-D-F1027, ICD-10-D-F10280, ICD-10-D-F10281, ICD-10-D-F10282, ICD-10-D-F10288, ICD-10-D-F10929, ICD-10-D-F10950, ICD-10-D-F10951, ICD-10-D-F10959, ICD-10-D-F1096, ICD-10-D-F10980, ICD-10-D-F10982, ICD-10-D-F1099, ICD-10-D-K700, ICD-10-D-K7010, ICD-10-D-K7030, ICD-10-D-K709, ICD-10-D-R780, ICD-10-D-Z1389, ICD-10-D-Z658  ICD-9-D-292, ICD-9-D-2920, ICD-9-D-2921, ICD-9-D-29211, ICD-9-D-29212, ICD-9-D-2922, ICD-9-D-2928, ICD-9-D-29281, ICD-9-D-29282, ICD-9-D-29283, ICD-9-D-29284, ICD-9-D-29285, ICD-9-D-29289, ICD-9-D-2929, ICD-9-D-304, ICD-9-D-3040, ICD-9-D-30400, ICD-9-D-30401, ICD-9-D-30402, ICD-9-D-30403, ICD-9-D-3041, ICD-9-D-30410, ICD-9-D-30411, ICD-9-D-30412, ICD-9-D-30413, ICD-9-D-3042, ICD-9-D-30420, ICD-9-D-30421, ICD-9-D-30422, ICD-9-D-30423, ICD-9-D-3043, ICD-9-D-30430, ICD-9-D-30431, ICD-9-D-30432, ICD-9-D-30433, ICD-9-D-3044, ICD-9-D-30440, ICD-9-D-30441, ICD-9-D-30442, ICD-9-D-30443, ICD-9-D-3045, ICD-9-D-30450, ICD-9-D-30451, ICD-9-D-30452, ICD-9-D-30453, ICD-9-D-3046, ICD-9-D-30460, ICD-9-D-30461, ICD-9-D-30462, ICD-9-D-30463, ICD-9-D-3047, ICD-9-D-30470, ICD-9-D-30471, ICD-9-D-30472, ICD-9-D-30473, ICD-9-D-3048, ICD-9-D-30480, ICD-9-D-30481, ICD-9-D-30482, ICD-9-D-30483, ICD-9-D-3049, ICD-9-D-30490, ICD-9-D-30491, ICD-9-D-30492, ICD-9-D-30493, ICD-9-D-3051, ICD-9-D-30510, ICD-9-D-30511, ICD-9-D-30512, ICD-9-D-30513, ICD-9-D-3052, ICD-9-D-30520, ICD-9-D-30521, ICD-9-D-30522, ICD-9-D-30523, ICD-9-D-3053, ICD-9-D-30530, ICD-9-D-30531, ICD-9-D-30532, ICD-9-D-30533, ICD-9-D-3054, ICD-9-D-30540, ICD-9-D-30541, ICD-9-D-30542, ICD-9-D-30543, ICD-9-D-3055, ICD-9-D-30550, ICD-9-D-30551, ICD-9-D-30552, ICD-9-D-30553, ICD-9-D-3056, ICD-9-D-30560, ICD-9-D-30561, ICD-9-D-30562, ICD-9-D-30563, ICD-9-D-3057, ICD-9-D-30570, ICD-9-D-30571, ICD-9-D-30572, ICD-9-D-30573, ICD-9-D-3058, ICD-9-D-30580, ICD-9-D-30581, ICD-9-D-30582, ICD-9-D-30583, ICD-9-D-3059, ICD-9-D-30590, ICD-9-D-30591, ICD-9-D-30592, ICD-9-D-30593, ICD-10-D-F1110, ICD-10-D-F11159, ICD-10-D-F11181, ICD-10-D-F11182, ICD-10-D-F11188, ICD-10-D-F1120, ICD-10-D-F1121, ICD-10-D-F11222, ICD-10-D-F11259, ICD-10-D-F11281, ICD-10-D-F11282, ICD-10-D-F11288, ICD-10-D-F11922, ICD-10-D-F11959, ICD-10-D-F11981, ICD-10-D-F11982, ICD-10-D-F11988, ICD-10-D-F1210, ICD-10-D-F12122, ICD-10-D-F12159, ICD-10-D-F12180, ICD-10-D-F12188, ICD-10-D-F1220, ICD-10-D-F1221, ICD-10-D-F12222, ICD-10-D-F12259, ICD-10-D-F12280, ICD-10-D-F12288, ICD-10-D-F1290, ICD-10-D-F12922, ICD-10-D-F12959, ICD-10-D-F12980, ICD-10-D-F12988, ICD-10-D-F1310, ICD-10-D-F13159, ICD-10-D-F13180, ICD-10-D-F13181, ICD-10-D-F13182, ICD-10-D-F13188, ICD-10-D-F1320, ICD-10-D-F1321, ICD-10-D-F13259, ICD-10-D-F13280, ICD-10-D-F13281, ICD-10-D-F13282, ICD-10-D-F13288, ICD-10-D-F13959, ICD-10-D-F13980, ICD-10-D-F13981, ICD-10-D-F13982, ICD-10-D-F13988, ICD-10-D-F1410, ICD-10-D-F14122, ICD-10-D-F14159, ICD-10-D-F14180, ICD-10-D-F14181, ICD-10-D-F14182, ICD-10-D-F14188, ICD-10-D-F1420, ICD-10-D-F1421, ICD-10-D-F14222, ICD-10-D-F14259, ICD-10-D-F14280, ICD-10-D-F14281, ICD-10-D-F14282, ICD-10-D-F14288, ICD-10-D-F14922, ICD-10-D-F14959, ICD-10-D-F14980, ICD-10-D-F14981, ICD-10-D-F14982, ICD-10-D-F14988, ICD-10-D-F1510, ICD-10-D-F15122, ICD-10-D-F15159, ICD-10-D-F15180, ICD-10-D-F15181, ICD-10-D-F15182, ICD-10-D-F15188, ICD-10-D-F1520, ICD-10-D-F1521, ICD-10-D-F15222, ICD-10-D-F15259, ICD-10-D-F15280, ICD-10-D-F15281, ICD-10-D-F15282, ICD-10-D-F15288, ICD-10-D-F15920, ICD-10-D-F15922, ICD-10-D-F15959, ICD-10-D-F15980, ICD-10-D-F15981, ICD-10-D-F15982, ICD-10-D-F15988, ICD-10-D-F1610, ICD-10-D-F16122, ICD-10-D-F16159, ICD-10-D-F16180, ICD-10-D-F16183, ICD-10-D-F16188, ICD-10-D-F1620, ICD-10-D-F1621, ICD-10-D-F16259, ICD-10-D-F16280, ICD-10-D-F16283, ICD-10-D-F16288, ICD-10-D-F16959, ICD-10-D-F16980, ICD-10-D-F16983, ICD-10-D-F16988, ICD-10-D-F17200, ICD-10-D-F17208, ICD-10-D-F17218, ICD-10-D-F17228, ICD-10-D-F17298, ICD-10-D-F1810, ICD-10-D-F18159, ICD-10-D-F18180, ICD-10-D-F18188, ICD-10-D-F18259, ICD-10-D-F18280, ICD-10-D-F18288, ICD-10-D-F18959, ICD-10-D-F18980, ICD-10-D-F18988, ICD-10-D-F1910, ICD-10-D-F19122, ICD-10-D-F19159, ICD-10-D-F19180, ICD-10-D-F19181, ICD-10-D-F19182, ICD-10-D-F19188, ICD-10-D-F1920, ICD-10-D-F1921, ICD-10-D-F19222, ICD-10-D-F19259, ICD-10-D-F19280, ICD-10-D-F19281, ICD-10-D-F19282, ICD-10-D-F19288, ICD-10-D-F19921, ICD-10-D-F19922, ICD-10-D-F19939, ICD-10-D-F1994, ICD-10-D-F19950, ICD-10-D-F19951, ICD-10-D-F19959, ICD-10-D-F1996, ICD-10-D-F1997, ICD-10-D-F19980, ICD-10-D-F19981, ICD-10-D-F19982, ICD-10-D-F19988, ICD-10-D-F1999 |
| **Antidepressants**  Selective Serotonin Reuptake Inhibitor (SSRI)  Selective Norepinephrine Reuptake Inhibitor (SNRI)  Tricyclic Antidepressants (TCA)  Atypical Antidepressants  Serotonin Modulators  Monoamine Oxidase Inhibitors (MAOi) | SERTRALINE_HCL, FLUVOXAMINE_MALETE, FLUOXETINE, FLUOXETINE_HCL, PAROXETINE_HCL, PAROXETEINE_MESYLATE, CITALOPRAM_HYDROBROMIDE, ESCITALOPRAM_OXALATE  VENLAFAXINE_HCL, VENLAFAXINE_BESYLATE, DESVENLAFAXINE, DESVENLAFAXINE_FUMARATE, DESVENLAFAXINE_SUCCINATE, DULOXETINE_HCL, LEVOMINACIPRAN_HCL, MINACIPRAN_HCL  AMITRIPTYLINE_HCL, AMITRIPTYLINE_HCL, AMITRIPTYLINE_CHLORDIAZEPOXIDE, CLOMIPRAMINE_HCL, DESIPRAMINE_HCL, IMIPRAMINE_HCL, IMIPRAMINE_PAMOATE, TRIMIPRAMINE_MALEATE, NORTRIPTYLINE_HCL, PROTRIPTYLINE_HCL, MAPROTILINE_HCL, AMOXAPINE  BUPROPION_HBR, BUPROPION_HCL, DEXTROMETHORPHAN_HBR/BUPROPION, MIRTAZAPINE  NEFAZODONE, TRAZODONE_HCL, VILAZODONE_HCL, VORTIOXETINE_HYDROBROMIDE  SELEGILINE, SELEGILINE_HCL, TRANYLCYPROMINE_SULFATE, PHENELZINE_SULFATE, ISOCARBOXAZID |
| **Psychotherapy** | CPT-90832, CPT-90834, CPT-90837, CPT-90839 |
